# Large-scale investigation of deep learning approaches for ventilated lung segmentation using multi-nuclear hyperpolarized gas MRI

**DOI:** 10.1038/s41598-022-14672-2

**Published:** 2022-06-22

**Authors:** Joshua R. Astley, Alberto M. Biancardi, Paul J. C. Hughes, Helen Marshall, Laurie J. Smith, Guilhem J. Collier, James A. Eaden, Nicholas D. Weatherley, Matthew Q. Hatton, Jim M. Wild, Bilal A. Tahir

**Affiliations:** 1 Department of Oncology and Metabolism, The University of Sheffield, Sheffield, UK; 2 POLARIS, Department of Infection, Immunity and Cardiovascular Disease, The University of Sheffield, Sheffield, UK; 3 Insigneo Institute for In Silico Medicine, The University of Sheffield, Sheffield, UK

**Keywords:** Magnetic resonance imaging, Respiratory tract diseases

## Abstract

Respiratory diseases are leading causes of mortality and morbidity worldwide. Pulmonary imaging is an essential component of the diagnosis, treatment planning, monitoring, and treatment assessment of respiratory diseases. Insights into numerous pulmonary pathologies can be gleaned from functional lung MRI techniques. These include hyperpolarized gas ventilation MRI, which enables visualization and quantification of regional lung ventilation with high spatial resolution. Segmentation of the ventilated lung is required to calculate clinically relevant biomarkers. Recent research in deep learning (DL) has shown promising results for numerous segmentation problems. Here, we evaluate several 3D convolutional neural networks to segment ventilated lung regions on hyperpolarized gas MRI scans. The dataset consists of 759 helium-3 (^3^He) or xenon-129 (^129^Xe) volumetric scans and corresponding expert segmentations from 341 healthy subjects and patients with a wide range of pathologies. We evaluated segmentation performance for several DL experimental methods via overlap, distance and error metrics and compared them to conventional segmentation methods, namely, spatial fuzzy c-means (SFCM) and K-means clustering. We observed that training on combined ^3^He and ^129^Xe MRI scans using a 3D nn-UNet outperformed other DL methods, achieving a mean ± SD Dice coefficient of 0.963 ± 0.018, average boundary Hausdorff distance of 1.505 ± 0.969 mm, Hausdorff 95th percentile of 5.754 ± 6.621 mm and relative error of 0.075 ± 0.039. Moreover, limited differences in performance were observed between ^129^Xe and ^3^He scans in the testing set. Combined training on ^129^Xe and ^3^He yielded statistically significant improvements over the conventional methods (p < 0.0001). In addition, we observed very strong correlation and agreement between DL and expert segmentations, with Pearson correlation of 0.99 (p < 0.0001) and Bland–Altman bias of − 0.8%. The DL approach evaluated provides accurate, robust and rapid segmentations of ventilated lung regions and successfully excludes non-lung regions such as the airways and artefacts. This approach is expected to eliminate the need for, or significantly reduce, subsequent time-consuming manual editing.

## Introduction

Respiratory diseases are leading causes of mortality and morbidity worldwide with 339 million experiencing asthma, 65 million people with chronic obstructive pulmonary disease (COPD)^1,2^ and 1.8 million new lung cancer cases diagnosed every year^3^. Pulmonary imaging, using various modalities, is an essential part of the diagnosis, treatment planning, monitoring, and treatment assessment of respiratory diseases. The acquisition, processing, and interpretation of pulmonary images are critical components of patient management and are essential in reducing mortality and morbidity.

Currently, computed tomography (CT) is the clinical gold standard for pulmonary imaging due to its exceptional spatial and temporal resolution, and its ubiquitous availability. CT is a structural imaging modality that provides exquisite detail of morphological changes in the lung parenchyma but employs ionizing radiation. Although proton magnetic resonance imaging (^1^H MRI) has historically been susceptible to the low proton density in lungs, recent advances in pulse sequences and hardware with ultra-short and zero echo times have enabled ^1^H MRI to compete with CT with the added benefit of no ionizing radiation^4,5^. However, whilst structural imaging modalities facilitate the assessment of changes in lung tissue density, they do not directly provide an accurate picture of regional lung function.

Although nuclear imaging modalities such as single-photon emission computed tomography (SPECT) can provide regional lung function information^6^, they require harmful ionizing radiation, reducing the ability to conduct regular scans during clinical care. This is particularly important when imaging children, as developing tissue is more sensitive to ionizing radiation. Moreover, SPECT is limited by poor temporal and spatial resolution and images acquired using ^99m^Tc-diethylenetriamine pentaacetate (DTPA) aerosols, one of the most commonly used radiotracers for ventilation imaging with SPECT, are subject to clumping artefacts^6,7^. In contrast, unparalleled insights into respiratory diseases can be gleaned from non-ionizing functional lung MRI modalities, such as dynamic contrast-enhanced lung perfusion MRI and hyperpolarized gas ventilation MRI. Hyperpolarized gas MRI provides visualization and quantification of regional lung ventilation with high spatial resolution within a single breath^8^. Quantitative biomarkers derived from this modality, including the ventilated defect percentage (VDP) and coefficient of variation, provide further insights into regional ventilation^9–11^. To facilitate the computation of such biomarkers, segmentation of ventilated regions of the lungs is required^12^.

Previous approaches for hyperpolarized gas MRI ventilation segmentation employed classical image processing and machine learning approaches, such as hierarchical K-means^13^ and spatial fuzzy c-means (SFCM) clustering^14^. However, as these methods rely on voxel intensities and thresholding, they only provide semi-automatic segmentations; as such, they are prone to generate errors in regions where voxel intensities are similar to those of the ventilated lung region (e.g., airways and artefacts). Consequently, they frequently require significant time to manually correct.

Deep learning (DL), which utilizes artificial neural networks with multiple hidden layers, has shown tremendous promise in medical image segmentation applications^15^. Although DL was initially theorized over half a century ago, the field only received widespread acclaim in 2012 when AlexNet, a form of an artificial neural network referred to as a convolutional neural network (CNN), triumphed in the ImageNet Large Scale Visual Recognition Challenge^16^. Subsequently, CNNs, and DL more generally, have become mainstream in the medical image segmentation field. UNet and VNet CNNs have demonstrated their profound impact in numerous medical image segmentation problems^17,18^. Adoption has been enhanced through transfer learning to cope with limited datasets common in the medical imaging field^19^. In a recent review of DL-based lung image analysis studies, Astley et al. identified a significant gap in DL-based lung MRI segmentation studies (n = 7) with only one published conference proceeding^20^ and one journal article^21^ evaluating DL for hyperpolarized gas MRI segmentation. Tustison et al. used a 2D UNet for hyperpolarized gas MRI segmentation on a dataset of 113 images, developing a novel template-based method to augment the limited lung imaging data alongside pre-processing techniques, including N4 bias correction and adaptive denoising. A mean ± SD DSC between DL and manual segmentations of 0.94 ± 0.03 was achieved^21^. However, the application of DL on a more extensive dataset with a broader range of pathologies is required prior to clinical adoption.

In this work we conducted extensive parameterization experiments to determine the best-performing 3D CNN architecture, loss function and pre-processing techniques for hyperpolarized gas MRI segmentation. We further evaluated five DL methods using the best performing configuration to accurately, robustly and rapidly segment ventilated lungs on hyperpolarized gas MRI scans. Using a diverse testing set, with both helium-3 (^3^He) and xenon-129 (^129^Xe) noble gas scans and corresponding expert segmentations, we evaluated and compared performance using a range of evaluation metrics. We also investigated the effect of the noble gas on DL performance. Furthermore, we compared the best performing DL method to conventional approaches. Finally, ventilated lung volume correlation and agreement were assessed for the best-performing DL method compared to expert-derived volumes.

## Materials and methods

### Hyperpolarized gas MRI acquisition

All subjects underwent 3D volumetric ^3^He or ^129^Xe hyperpolarized gas MRI with full lung coverage at 1.5 T on a HDx scanner (GE Healthcare, Milwaukee, WI) using 3D steady-state free precession (SSFP) sequences as previously described^22–24^. Flexible quadrature radiofrequency coils were employed for transmission and reception of MR signals at the Larmor frequencies of ^3^He and ^129^Xe. In-plane (x–y) resolution of scans for both gases was 4 × 4mm^2^. ^129^Xe scans ranged from 16 to 34 slices with a mean of 23 slices and slice thickness of 10 mm. ^3^He scans ranged from 34 to 56 slices with a mean of 45 slices and slice thickness of 5 mm.

### Dataset

The imaging dataset used in this study was pooled retrospectively from several research studies and clinical studies of patients referred for hyperpolarized gas MRI scans. Data use was approved by the Institutional Review Boards at the University of Sheffield and the National Research Ethics Committee. All data was anonymized and all investigations were conducted in accordance with the relevant guidelines and regulations.

The dataset consisted of 759 volumetric hyperpolarized gas MRI scans (23,265 2D slices), with either ^3^He (264 scans, 11,880 slices) or ^129^Xe (495 scans, 11,385 slices), from 341 subjects. The slices were distributed approximately 50:50 between ^3^He and ^129^Xe. The dataset contained healthy subjects and patients with various pulmonary pathologies: asthma, COPD, asthma/COPD overlap, bronchiectasis, interstitial lung disease (ILD), idiopathic pulmonary fibrosis (IPF), lung cancer, cystic fibrosis (CF), children born prematurely, and patients investigated for possible airway disease. Demographic and clinical data for these subjects are summarized in Table 1.

**Table 1:** Summary of demographics, clinical characteristics and image dataset information stratified by disease.

Disease	Total number of scans	Number of patients	Number of HP gas scans		Sex*		Median (range) age*	Mean ± SD ventilated lung volume (liters)*
			^3^He	^129^Xe	Male	Female		
Healthy	43	33	1	42	15	13	12 (9, 76)	3.78 ± 1.18
Asthma	169	81	4	165	28	52	50 (13, 73)	4.23 ± 1.03
Asthma/COPD overlap	11	5	0	11	0	5	56 (45, 67)	4.13 ± 0.68
Bronchiectasis	3	3	1	2	1	1	15 (9, 29)	3.76 ± 1.00
CF	247	58	134	113	29	28	16 (6, 48)	3.65 ± 1.05
COPD	62	23	56	6	4	5	64 (52, 80)	4.43 ± 0.71
Non-IPF ILD**	77	41	0	77	25	16	69 (39, 83)	3.78 ± 0.80
Investigation for possible airways disease	38	21	5	33	2	16	49 (36, 69)	3.89 ± 1.05
IPF	46	20	45	1	17	3	72 (52, 80)	3.87 ± 0.71
Lung cancer	22	16	14	8	10	6	69 (34, 85)	4.12 ± 0.86
Preterm birth	41	40	4	37	15	25	12 (9, 14)	2.75 ± 0.55

*HP* hyperpolarized, *CF* cystic fibrosis, *COPD* chronic obstructive pulmonary disease, *ILD* interstitial lung disease, *IPF* idiopathic pulmonary fibrosis, *SD* standard deviation.

*Data for 25 patients was unavailable.

**Contains connective tissue disease-associated ILD (CTD-ILD), hypersensitivity pneumonitis and drug-induced ILD (DI-ILD).

Each of the 759 scans in the dataset has a corresponding, manually-edited expert segmentation, representing the ventilated region of the lungs. These scans and segmentations were collected from numerous retrospective studies; consequently, the segmentations were generated using several semi-automated methods^14,25^ and edited by multiple expert observers. Quality control was conducted by an experienced imaging scientist who identified potential errors and manually corrected them to ensure segmentation accuracy; the airways were removed down to the third generation, and it was ensured that no voxels were outside of the lung parenchymal region defined by a structural ^1^H MRI scan, thereby removing background noise.

### Convolutional neural network

**Figure 1:**
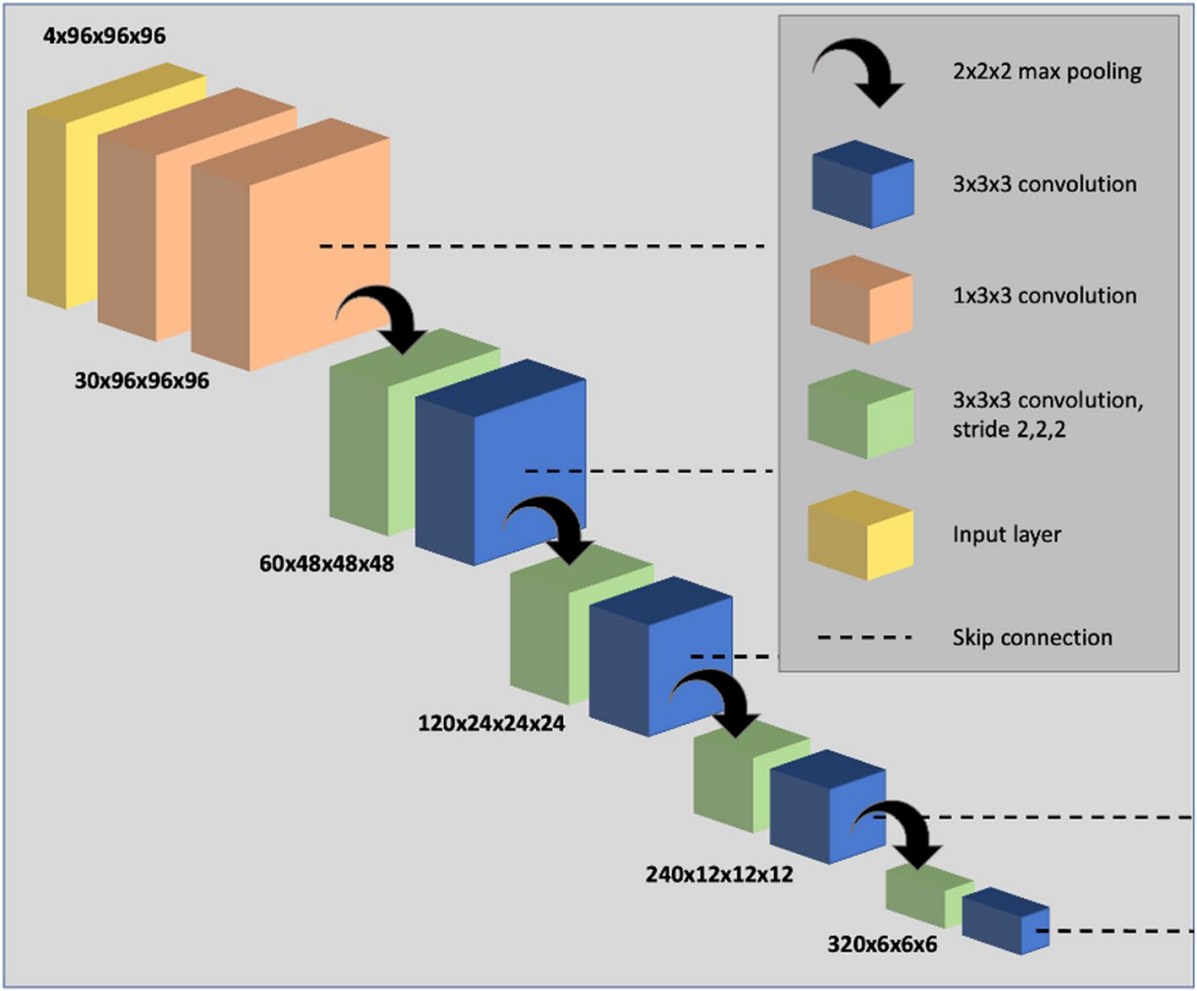
Visual representation of the modified 3D nn-UNet network used in this work. The deconvolution side of the network is omitted as it follows the same structure as the convolutional path, however, with the addition of a 1 × 1 × 1 SoftMax layer.

Parameterization experiments were conducted comparing network architectures, loss functions and pre-processing techniques; results of these experiments are provided in the Supplementary Information. We used the nn-UNet fully convolutional neural network which processes 3D scans using volumetric convolutions. The network is trained end-to-end using hyperpolarized gas MRI volumetric scans. We use a 3D implementation of the UNet which has been modified to reduce memory constraints, allowing 30 feature channels^26^. Convolution operations vary in size from 3 × 3 × 3 to 1 × 1 × 1 depending on the layer of the network. The network also makes use of instance normalization. An isotropic spatial window size was used of [96, 96, 96] with a batch size of 2. A high-level visual representation of the 3D nn-UNet, specific to the spatial window sizes used, is shown in Fig. 1.

#### Parameters

The network utilizes a non-linear PReLU activation function^27^ and is optimized using a binary cross-entropy loss function. ADAM optimization was used to train the CNN^28^ and instance normalization was conducted for each pass. The spatial window size was [96, 96, 96] with a batch size of 2. A learning rate of 1 × 10^−5^ was used for initial training and 0.5 × 10^−5^ for subsequent fine-tuning methods.

#### Pre-processing

Each hyperpolarized gas MRI scan was pre-processed using spatially adaptive denoising, designed to consider both Rician noise and spatially varying patterns of noise. Denoising was implemented with ANTs 2.1.0 using the DenoiseImage function across three dimensions. Standard parameters were used^29^.

#### Data augmentation

Constrained random rotation and scaling was used for data augmentation. Rotation with limits − 10° to 10° and scaling of − 10 to 10%, where a random rotation or scaling were applied at an interval within those limits, were used. A different random value was computed for each rotation axis and scaling factor.

#### Data split

The dataset was randomly split into training and testing sets with 75% and 25% of the data respectively, in terms of the number of subjects. The training set, therefore, contained 237 ^3^He scans (10,902 slices) and 436 ^129^Xe scans (10,028 slices) from a total of 255 subjects. 86 scans, each from a different subject, were selected for the testing set (^3^He: 27 scans (1242 slices); ^129^Xe: 59 scans (1357 slices)). Repeat or longitudinal scans from multiple visits for the same patient were contained in the training set; however, no subject was present in both the training and testing sets, with the testing set containing only one scan from each patient. This was ensured by randomly selecting only one scan from each subject in the testing set and discarding the remaining scans; these scans are not included in Table 1. The range of diseases in the testing set was representative of the dataset as a whole. In addition, it was specified that there would be no overlap between the newly defined testing set and the previous testing set used for parameterization experiments, described in the Supplementary Information, in terms of either patient or scan.

#### Computation

All networks were trained using the medical imaging DL framework NiftyNet 0.6.0^30^ built on top of TensorFlow 1.14^31^. Training and inference were performed on an NVIDIA Tesla V100 GPU with 16 GB of RAM.

### DL experimental methods

Five DL experimental methods were performed to train the network:

1. The model was trained on 237 ^3^He scans for 30,000 iterations.
2. The model was trained on 436 ^129^Xe scans for 30,000 iterations.
3. The model was trained on 237 ^3^He scans for 20000 iterations; these weights were used to initialize a model trained on 436 ^129^Xe scans for 10000 iterations.
4. The model was trained on 436 ^129^Xe scans for 20000 iterations; these weights were used to initialize a model trained on 237 ^3^He scans for 10000 iterations.
5. The model was trained on 436 ^129^Xe and 237 ^3^He scans for 30,000 iterations.

The five experimental methods were applied to the data split defined above using the same testing set for each method, facilitating comparison between the five methods to identify the best performing training method across multiple metrics.

### Comparison to conventional methods

For further benchmarking, the best-performing DL method was compared against two other conventional machine learning methods, namely, SFCM and K-means clustering. The methods used are described as follows:

1. The k-means clustering algorithm used here was previously modified for hyperpolarized gas MRI segmentation^32^. This method attempts to find k data points, given the integer k, in an n-dimensional space (R^n^) given m data points. These k data points are known as centres/centroids and the aim is to minimize the distance from each data point (m) to its centre/centroid^33^. The previously developed method^32^ attempts to delineate the image data into a number of clusters that can best represent a radiologist’s analysis of the ventilation image with clusters defined from defects to hyperintense signal. The first stage of this method requires image normalization into the range of 0–255, following which the cluster initial centers are set at 25% intervals between these values. A two-stage clustering process was applied with four clusters in the first stage, the lowest of which contains both signal void and hypointense signal. In the second stage, the clustering was reapplied to the lowest cluster from the first stage to define background, ventilation defect and hypointense signal regions.
2. The SFCM method used in this work has been reported previously^14^; images are initially filtered to remove noise and maintain edges using a bilateral filter^34^. The standard FCM algorithm assigns *N* pixels to *C* clusters via Fuzzy memberships. The key assumption of the Spatial Fuzzy C-means is that pixels spatially close will have high correlation and hence have similarly high membership to the same cluster. This spatial information will modify the membership value only if, for example, the pixel is noisy and would have been incorrectly classified. The SFCM method makes use of nearby pixels during the iteration process by taking into account the membership of voxels within a predefined window (5×5 in this work) and will weight the central pixel depending on the provided weighting variables^35^. The optimal number of clusters was manually selected by the observer.

### Evaluation Metrics

The testing set results for each of the five DL experimental methods and two conventional methods were evaluated using several metrics. The DSC was used to assess overlap between the ground truth *(GT)* and predicted *(PR)* segmentations^36^ and is defined as:

1 $$ DSC = 2\frac{{\left| {PR \cap GT} \right|}}{{\left| {PR} \right| + \left| {GT} \right|}} $$

Two distance metrics, average boundary Hausdorff distance (Avg HD) and 95th percentile Hausdorff distance (HD95) were used^37^ and are defined as the following:

2 $$HD(PR,GT)=\max(h(PR,GT),h(GT,PR))$$

where *h*(*PR*, *GT*) represents the directed Hausdorff distance between the sets of *PR* and *GT* voxels at the boundary, *pr* represents an individual voxel in the set *PR* and *gt* represents an individual boundary voxel in *GT*. *h*(*PR*, *GT*) is defined as:

3 $$ {\text{h}}\left( {\text{PR,GT}} \right) = \mathop {\max }\limits_{pr \in PR} \mathop {\min }\limits_{gt \in GT} \|PR - GT\| $$

$\left\| {PR - GT} \right\|$ is the Euclidean distance between *PR* and *GT*. From this, HD95 is calculated as the 95th percentile of Eq. (2) and is frequently used in the image segmentation literature to remove the impact of outlier voxels. The Avg HD is defined similarly as:

4 $$ Avg~HD\left( {PR,GT} \right) = \max (d\left( {{\text{PR}},{\text{GT}}} \right),{\text{~d}}\left( {{\text{GT}},{\text{PR}})} \right)$$

where *d*(*PR*, *GT*) represents the directed average Hausdorff distance given by:

5 $$ {\text{d}}\left( {\text{PR,GT}} \right) = \frac{1}{N} \mathop \sum \limits_{pr \in PR} \mathop {\min }\limits_{gt \in GT} \|PR - GT\| $$

where N is the set of paired voxels in (*GT*, *PR*). The Avg HD reduces sensitivity to outliers and is regarded as a stable metric for segmentation evaluation^38^.

Furthermore, a relative error metric (XOR) was used to evaluate segmentation errors^39^ as follows:

6 $$ XOR = \frac{{\left| {PR \cap GT^{{\prime }} } \right| + \left| {PR^{{\prime }} \cap GT} \right|}}{{\left| {GT} \right|}} $$

where PR’ and GT’ are the complements of PR and GT, respectively. The metric was used specifically because it is expected to correlate with the manual editing time required to correct the segmentation outcome.

### Statistical analysis

Data were tested for normality using Shapiro–Wilk tests; when normality was not satisfied, non-parametric tests were conducted. One-way repeated-measure ANOVA or Friedman tests were conducted as appropriate with Bonferroni correction for post-hoc multiple comparisons to assess statistical significances of differences between experimental DL-based methods. Independent t-tests or Mann–Whitney U tests were used to compare differences between ^3^He and ^129^Xe segmentations in the testing set, assessing the effect of the noble gas. The best performing DL method was compared to other conventional segmentation methods using one-way repeated-measure ANOVA or Friedman tests with Bonferroni correction for post-hoc multiple comparisons. Pearson or Spearman correlations and Bland–Altman analysis were conducted to compare volumes of DL-generated and expert segmentations. Statistical analysis was performed using Prism 8.4 (GraphPad, San Diego, CA) and SPSS Statistics 26.0 (IBM Corporation, Armonk, NY).

## Results

**Figure 2:**
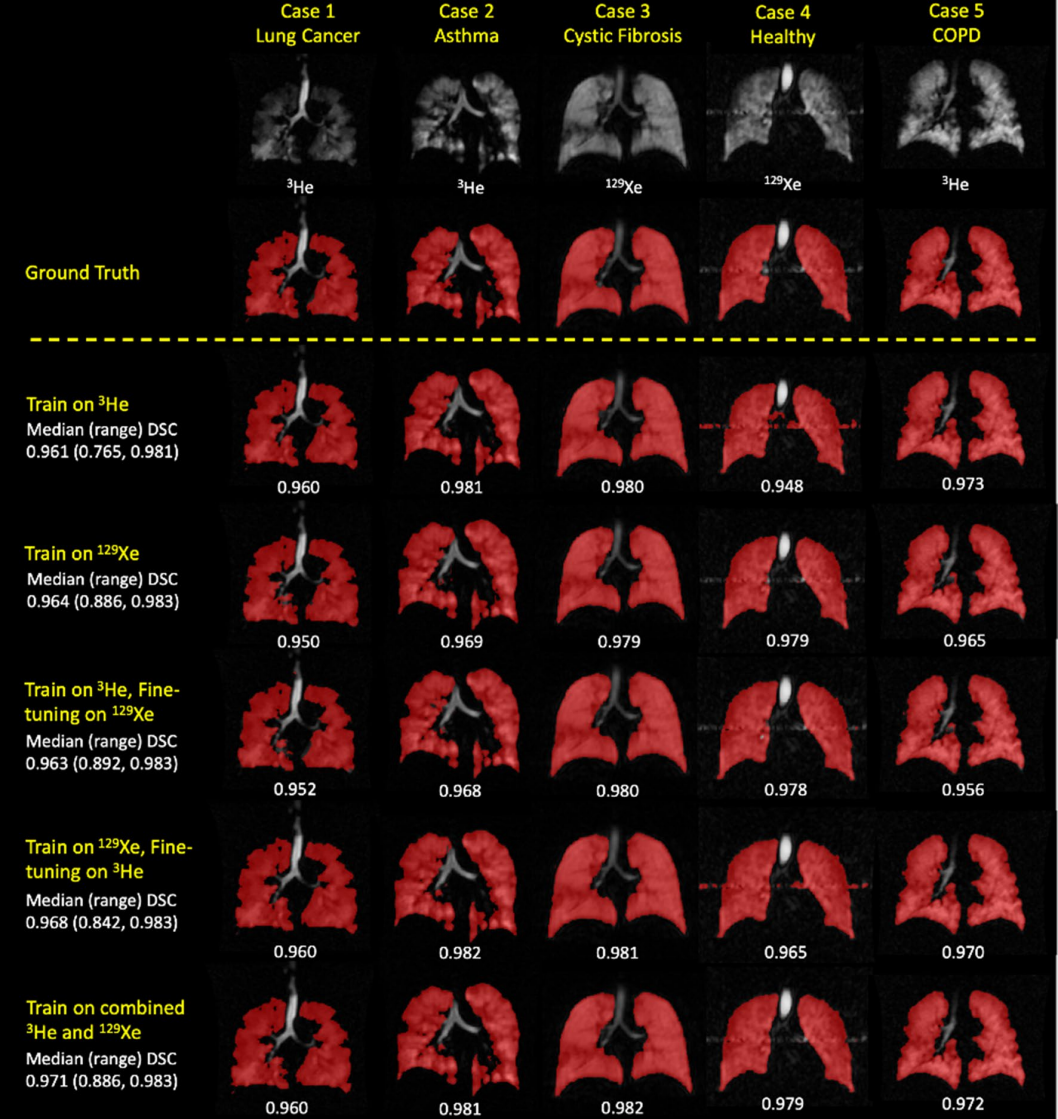
Example coronal slices for a healthy subject and four cases with different pathologies for each DL experimental method. Individual, and median (range), DSC values are displayed.

Segmentations for each of the five DL methods were generated for 86 testing set scans. Figure 2 shows examples of segmentation quality for a healthy subject and patients with four different pathologies across the five DL experimental methods using ^3^He or ^129^Xe. The original scans and expert segmentations are included to facilitate comparison. It can be observed that, in general, there are negligible voxels outside the lung parenchyma classed as ventilated and that the CNNs accurately excluded ventilation defects, as shown in the examples of the asthma and lung cancer patients. Case 4, of a healthy subject, represents an interesting case due to the presence of a zipper artefact caused by electronic noise in the hardware; it can be observed that some models are able to accurately exclude this artefact, whilst others remain unable to distinguish between the zipper artefact and ventilated lung voxels. Table 2 summarizes segmentation performance for the five DL experimental methods. The Combined ^3^He and ^129^Xe method generated the best segmentations using all four metrics.

**Table 2:** Comparison of segmentation performance for the five DL training methods for all scans in the testing set.

Experimental DL methods	Evaluation metrics: median (range)			
	DSC	Avg HD (mm)	HD95 (mm)	XOR
Train on ^3^He	0.961 (0.765, 0.981)	2.335 (35.91, 0.644)	10.00 (140.9, 1.934)	0.079 (0.613, 0.037)
Train on ^129^Xe	0.964 (0.886, 0.983)	1.341 (3.911, 0.675)	4.809 (15.90, 1.875)	0.072 (0.253, 0.035)
Train on ^3^He, fine-tuned on ^129^Xe	0.963 (0.892, 0.983)	1.384 (4.628, 0.636)	4.971 (29.80, 1.934)	0.075 (0.238, 0.034)
Train on ^129^Xe, fine-tuned on ^3^He	0.968 (0.842, 0.983)	1.483 (10.84, 0.596)	4.935 (67.85, 1.563)	0.066 (0.372, 0.034
Combined ^3^He and ^129^Xe training	**0.971 (0.886, 0.983)**	**1.234 (5.630, 0.594)**	**4.193 (52.70, 1.875)**	**0.059 (0.255, 0.035)**

Medians (ranges) are given; the best result for each metric is in bold.

**Figure 3:**
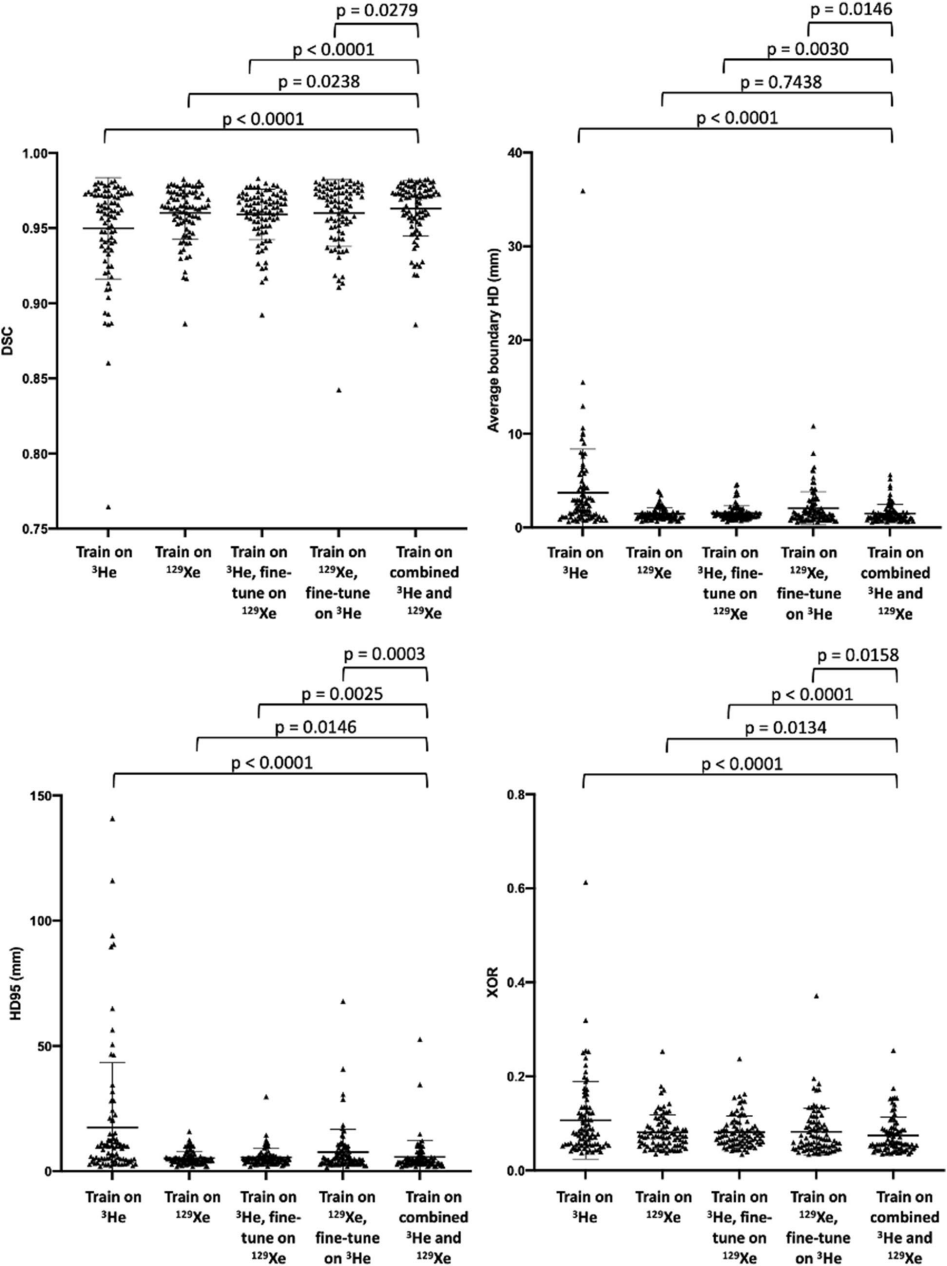
Comparison of segmentation performance on 86 testing scans for five DL experimental methods using the DSC, Avg HD, HD95 and XOR metrics (left to right). P-values are displayed for Friedman tests with Bonferroni correction for multiple comparisons, comparing the combined ^3^He and ^129^Xe DL method to the other DL methods. Mean and standard deviation values are marked by a bold line and whiskers, respectively.

Figure 3 shows distributions of all four metrics for each DL method. The assumption of normality for each metric was not satisfied for all DL methods, as assessed by Shapiro–Wilk’s tests (p < 0.05). As such, Friedman tests were run, determining that there were differences between DL methods for each metric. Post-hoc pairwise comparisons were performed for each metric with Bonferroni correction for multiple comparisons. The combined ^3^He and ^129^Xe method yielded statistically significant improvements over all DL methods using the DSC, XOR and HD95 metrics (p < 0.05). However, using the Avg HD metrics, the combined ^3^He and ^129^Xe method significantly outperformed all but one DL method.

**Figure 4:**
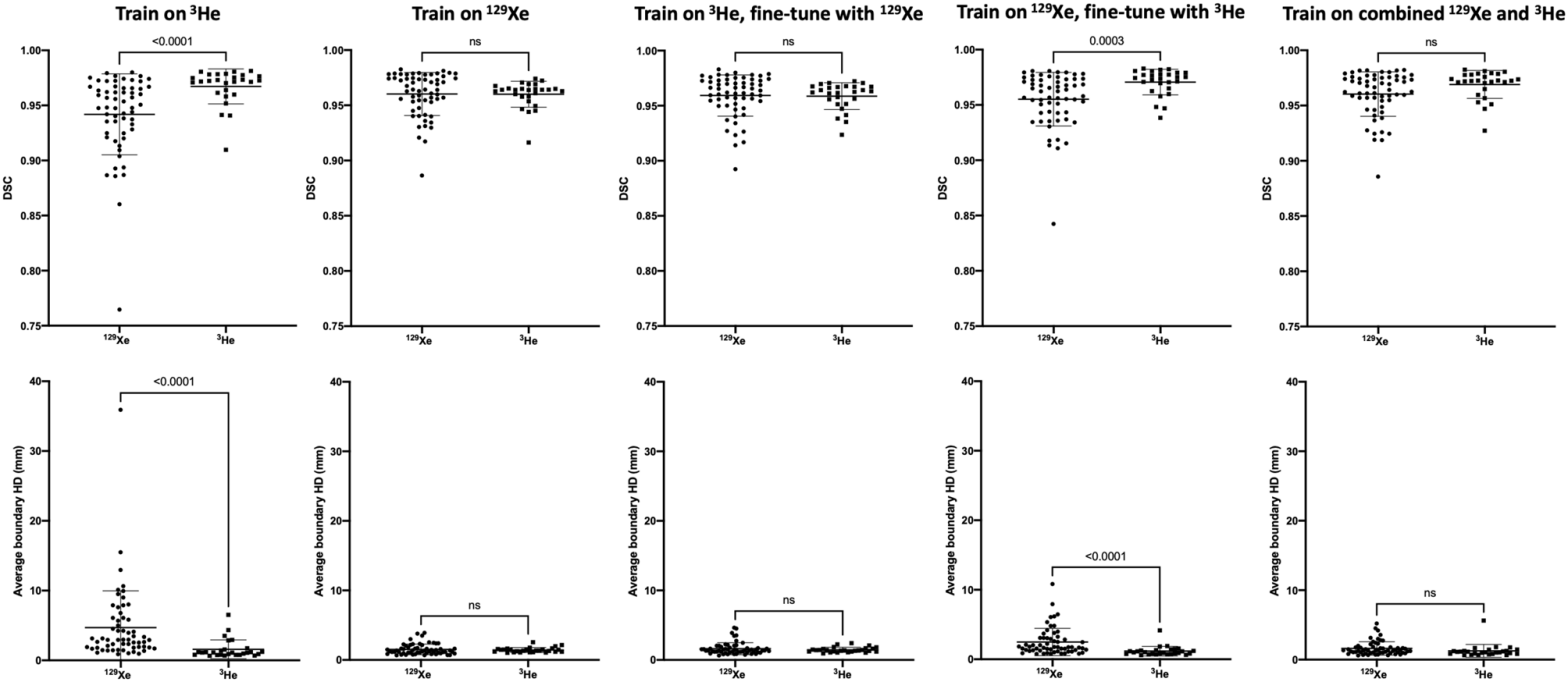
Comparison of DSC (top) and Avg HD (bottom) values for ^129^Xe and ^3^He testing scans for five DL methods. P-values between ^129^Xe and ^3^He using Mann–Whitney tests are shown. Mean and standard deviation values are marked by a bold line and whiskers, respectively.

Figure 4 shows the segmentation performance for the testing set stratified by noble gas (^129^Xe or ^3^He) using the DSC and Avg HD metrics. The majority of models show no significant difference between ^129^Xe and ^3^He for both metrics. Only two methods, namely, the ‘Train on ^3^He’ and ‘Train on ^129^Xe, fine-tune on ^3^He’ methods, showed a significant difference between noble gases across both metrics.

Based on the results of the five experimental methods, the combined ^3^He and ^129^Xe DL model was identified as the most accurate DL ventilated lung segmentation method due to statistically significant improvements over all other methods using the DSC, HD95 and XOR metrics. Consequently, we tested the combined ^3^He and ^129^Xe DL model on 31 2D spoiled gradient-echo ^3^He hyperpolarized gas MRI ventilation scans which differ in MRI sequence and acquisition parameters (see Supplemental Results). The results indicated that the model generalized to scans acquired with a different MRI sequence and produced high quality segmentations invariant of the sequence used.

**Figure 5:**
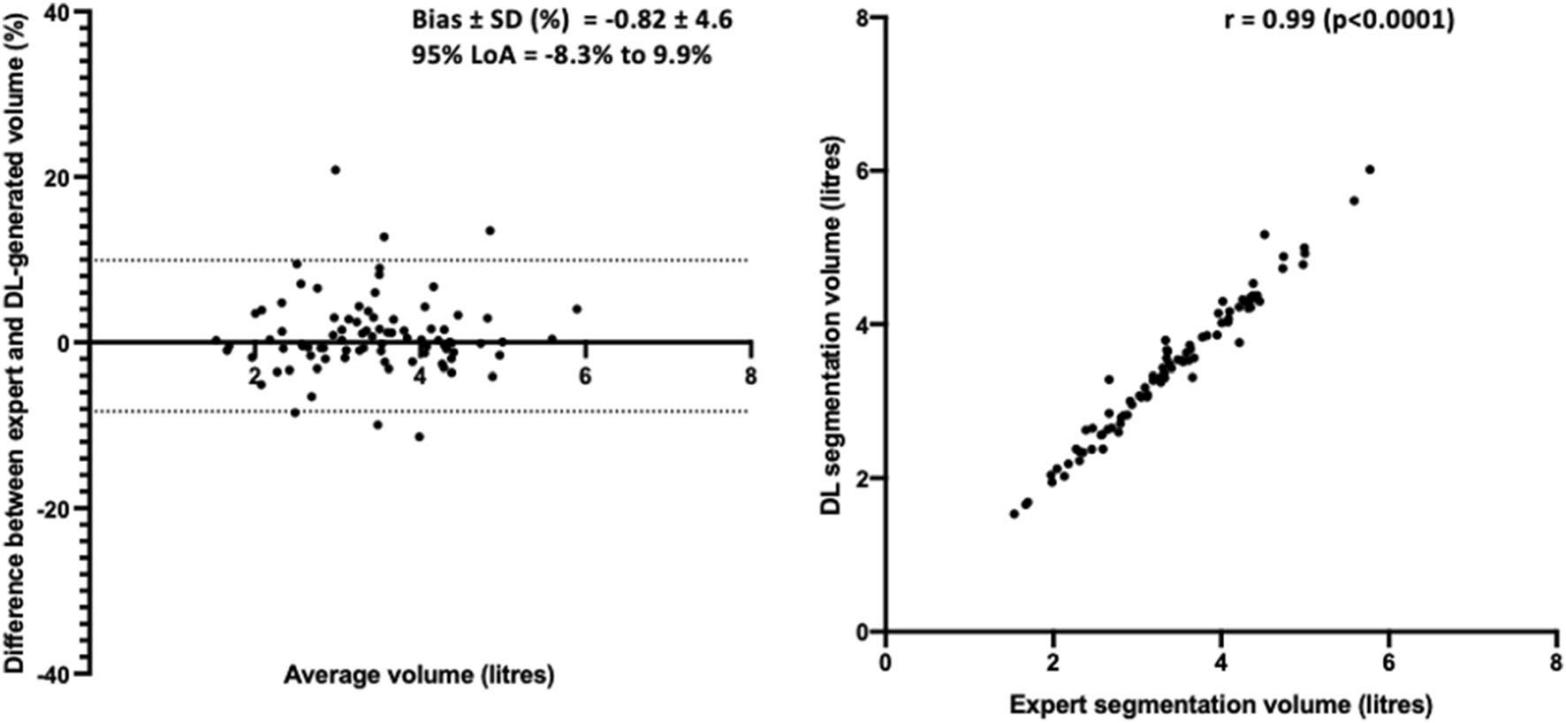
Pearson correlation and Bland–Altman analysis of lung volumes for 86 testing set cases compared to volumes derived from expert segmentations for the combined ^3^He and ^129^Xe DL model.

Furthermore, ventilated volume was assessed for the combined ^3^He and ^129^Xe method. The assumption of normality of was satisfied for DL and expert ventilated volume, as assessed by Shapiro–Wilk’s tests (p > 0.05). Pearson correlation and Bland–Altman analysis are shown in Fig. 5 for the combined ^129^Xe and ^3^He model; the DL segmentation volume is highly correlated (r = 0.99) with the expert segmentation volume and exhibits minimal bias (− 0.8%).

**Figure 6:**
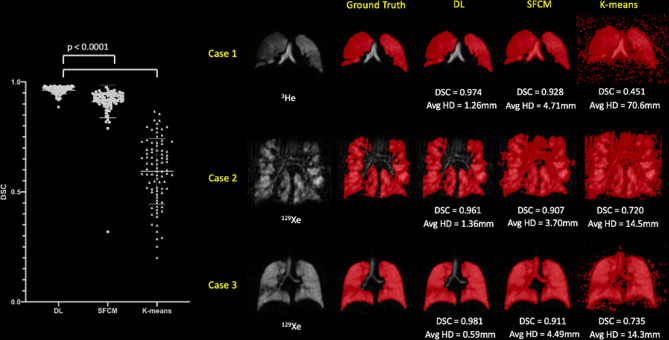
Comparison of performance on testing scans between the combined ^129^Xe and ^3^He DL method and conventional segmentation methods (SFCM and K-means) with P-values for Friedman tests with Bonferroni correction for multiple comparisons. Mean and standard deviation values are marked by a bold line and whiskers, respectively. Individual DSC and Avg HD values for each method are displayed for three cases.

Figure 6 shows qualitative and quantitative performance for the DL combined ^3^He and ^129^Xe training method with two conventional segmentation methods, namely K-means clustering and SFCM across three cases. The assumption of normality for the DSC metric was not satisfied for conventional and DL approaches, as assessed by Shapiro–Wilk’s tests. Post hoc Friedman’s tests were performed with Bonferroni correction for multiple comparisons (X^2^(3), p < 0.0001). The DL segmentation method exhibited significant improvements over conventional methods (p < 0.0001), accurately excluding low-level noise and artefacts (e.g. Case 2) as well as non-lung regions such as the trachea and bronchi.

## Discussion

The DL segmentation methods yielded highly accurate segmentations across a range of evaluation metrics on the dataset used. To the best of the authors’ knowledge, the hyperpolarized gas MRI dataset used here is the largest to date for ventilated lung segmentation, comprising 759 scans from patients with a wide range of lung pathologies. This is advantageous for preserving generalizability as it enables algorithms to learn features present in a range of diseases independent of the noble gas. Compared with ^129^Xe MRI, ^3^He MRI has an intrinsically stronger MRI signal due to the difference in gyromagnetic ratios between the two nuclei. Generally, lung ventilation information of similar diagnostic quality has been obtained with the two nuclei; despite this, there are known differences in lung diffusivity as well as differences in spatial resolution between the nuclei^40,41^. This is particularly important for deep learning applications as the resolutions of our ^3^He and ^129^Xe MRI scans differ greatly in the z-direction whereby ^3^He and ^129^Xe MRI scans have a slice thickness and an inter-slice distance of ~ 5 mm and ~ 10 mm, respectively. Therefore, it remains important to understand the performance of deep learning segmentation applications across the two nuclei.

The combined ^3^He and ^129^Xe DL method showed statistically significant improvements over all other methods using the DSC, HD95 and XOR metrics; however, using the Avg HD metric, no significant difference between the combined ^3^He and ^129^Xe method and ^129^Xe only method was observed, perhaps attributable to an outlier case. Some statistically significant differences were observed in performance when comparing ^3^He and ^129^Xe testing set scans; however, the combined ^3^He and ^129^Xe method exhibited identical performance independent of the noble gas used. This indicates that, for a given ^3^He or ^129^Xe scan, the combined ^3^He and ^129^Xe method is unlikely to be biased towards a specific noble gas. Due to the current paucity and unpredictable supplies of ^3^He worldwide, the field, in general, has transitioned towards the use of ^129^Xe as the predominant noble gas for hyperpolarized gas MR ventilation imaging. As this trend continues, it may be pertinent in future work to assess the impact of training and testing solely on ^129^Xe scans. In addition, external testing, detailed in the Supplemental Results, indicated the proposed model’s ability to generalize across MRI sequence and acquisition parameters not seen in the training set, further reinforcing that the model is using functional features from hyperpolarized gas MRI to produce accurate segmentations.

The CNN produced more accurate segmentations than the two conventional approaches for all evaluation metrics. In particular, the CNN was able to deal with images containing background noise and artefacts, as well as successfully excluding ventilation defects and airways. In comparison, the SFCM method was unable to distinguish airways or artefacts and segmented these areas erroneously. As such, it is highly probable that the CNN eliminates or dramatically reduces the manual-editing time required after automatic segmentation. The K-means clustering algorithm exhibited poorer than expected performance, possibly attributable to the lack of an available proton MRI. This represents a benefit of the CNN-based method as only the hyperpolarized gas MRI scan is required as an input. Previous work in the literature that aimed to employ DL for hyperpolarized gas MRI segmentation used a 2D UNet and achieved a mean DSC of 0.94^21^. In comparison, our combined ^3^He and ^129^Xe method trained via a 3D nn-UNet yielded a mean DSC value of 0.96. The 3D CNN allows the model to treat the segmentation as a 3D volume and learns features present across multiple slices e.g. ventilation defects. Several pre-processing techniques have previously been used in the literature for lung image segmentation^42^. The work of Tustison, et al.^21^ utilizes a novel template-based data augmentation strategy with N4 bias correction and denoising, which are computationally expensive and time-consuming; however, the impact of such techniques is not assessed in their work. In this study, we observed that N4 bias correction provided no significant benefit, while denoising yielded significant improvements.

All DL methods were trained and tested using a single GPU. Training required approximately nine days, while inference took 27 s per ^129^Xe scan and 35 s per ^3^He scan, corresponding to approximately one second per slice for both gases. Compared with conventional methods, such as SFCM, the time taken to generate automatic segmentations is significantly reduced from approximately 5 min to around 30 s, indicating the time-saving benefits of DL-based methods. Moreover, accurate automatic segmentation of hyperpolarized gas MRI ventilation scans through CNN-based approaches will eliminate or reduce manual editing time, thus improving clinical throughput. To further improve clinical translation of DL-based techniques, we have provided the trained DL model along with necessary files and Supplementary Instructions, enabling members of the pulmonary imaging community to apply the trained model in their own research.

The specific dataset used is unique within the context of pulmonary imaging due to the presence of numerous features such as different noble gases, longitudinal scans, repeat scans and pre- and post-treatment scans. The variation in the number of repeat or longitudinal scans and slice thicknesses between 3D ^3^He and ^129^Xe scans impeded us from achieving a training and testing set split equally between both gases; notwithstanding, the number of 2D slices were approximately equal between gases. Although multiple scans from the same patient were included in the training set to increase dataset numbers, to enhance the robustness of the evaluation, no scan of the same patient was present both in the training and testing sets.

This study also represents the first large-scale investigation of architectures, loss functions and pre-processing techniques within the field of lung MRI. Selecting a subset of the data allowed us to perform parameterization experiments to determine the ideal choice of network architecture, loss function and pre-processing technique, without creating optimization biases in subsequent experiments (see Supplementary Information). The conclusions of the parameterization experiments were partially limited due to multiple factors; the same exact parameters cannot be used for each network due to the spatial imaging constraints of the specific network, such as requiring isotropic resolutions or the varying memory requirements of each architecture. This means that the windowing, batch size and bordering varies between architectures and can, therefore, make comparisons potentially difficult. However, where possible, we aimed to maintain consistent parameters across all networks tested. Further investigation may aim to optimize other hyperparameters that could be deemed equally important as the experiments conducted related to architecture, loss function and pre-processing; these may include the choice of activation function or optimization algorithm. Furthermore, parameterization results will vary based on the specific datasets used and, consequently, limit conclusions to the particular data used in these experiments.

Currently, segmentations edited by expert observers are the gold-standard for training supervised DL algorithms. Studies have shown that manual segmentations are susceptible to inter-observer variability^43^. Numerous research projects have employed techniques to create generalizable DL models across multiple institutions and observers^44^. A limitation of our study is the presence of only one expert segmentation per scan, which precludes the ability to evaluate intra- and inter-observer variability. However, the wide range of expert observers used to generate and manually edit the expert segmentations led to significant variability in the training and testing sets. Hence, the CNN can learn a robust segmentation method invariant to the specific semi-automated method used to generate the ground truth or the expert observer who manually corrected it. In future work, multiple expert segmentations may be used to train the algorithm and allow the evaluation of inter-observer variability.

For the evaluation of certain clinically relevant metrics such as VDP^9^, the whole-lung cavity volume is required in addition to ventilated lung volumes, most commonly computed from a whole-lung segmentation generated from a structural proton MRI scan. In this work, we showed that ventilated lung volumes derived from CNN-generated segmentations have a significant Pearson correlation of 0.99 and a minimal Bland–Altman bias of − 0.8% with expert volumes. However, evaluation of DL-based methods using not only ventilated lung volume, but also VDP, would further the extensive validation required for clinical adoption.

## Conclusion

In conclusion, we evaluated a 3D fully connected CNN using the nn-UNet architecture that is capable of producing accurate, robust and rapid hyperpolarized gas MRI segmentations on a large, diverse dataset. We compared five experimental DL methods and observed that combining ^3^He and ^129^Xe scans during training produces significantly improved segmentations. Compared with expert segmentations, this CNN-based method also showed a strong Pearson correlation and limited bias using Bland–Altman analysis. In addition, the CNN-based segmentation method significantly outperformed two conventional segmentation methods commonly used in the literature.

## Supplementary Information

Supplementary Information.

## Data Availability

The imaging datasets generated and/or analysed during the current study are not publicly available as they were generated as part of an industrial collaborative study that is still underway. Requests for data should be addressed to J.M.W.
