# Supplementary material for: Large-scale investigation of deep learning approaches for ventilated lung segmentation using multi-nuclear hyperpolarized gas MRI
Source: Sci Rep. 2022 Jun 22;12:10566. doi: 10.1038/s41598-022-14672-2 (PMC9217976; doi:10.1038/s41598-022-14672-2)
Supplement: Supplementary file 1 — Supplementary Information. [file 41598_2022_14672_MOESM1_ESM.docx]

**Large-scale investigation of deep learning approaches for ventilated lung segmentation using multi-nuclear hyperpolarized gas MRI**

Joshua R. Astley^1,2^, Alberto M Biancardi^2^, Paul J.C. Hughes^2^, Helen Marshall^2^, Laurie J. Smith^2^, Guilhem J. Collier^2^, James A. Eaden^2^, Nicholas D. Weatherley^2^, Matthew Q. Hatton^1^, Jim M. Wild^2,3^, Bilal A. Tahir^1,2^

*^1^Department of Oncology and Metabolism, University of Sheffield, UK*

*^2^POLARIS, Department of Infection, Immunity & Cardiovascular Disease, University of Sheffield, UK*

*^3^Insigneo Institute for in silico medicine, University of Sheffield, UK*

**Supplemental Methods**

**Parameterization**

Parameterization is rarely conducted in the DL literature due to its time-consuming nature, computational cost and the possibilities of biasing future investigations using the same dataset. To this end, we used a subset of the data as the parameterization dataset comprising 431 hyperpolarized gas MRI scans (55% of the total data), with either ^3^He (n=173) or ^129^Xe (n=258) from a subset of the diseases present in the total dataset. 29 scans were used for the parameterization testing set. In this study, we conducted three distinct parameterization experiments that were completed sequentially; the outcome of the previous experiment influenced the inputs used in the subsequent experiment. We assessed the following hyperparameters:

***Fully convolutional network architecture -*** The network architecture concerns the structure of the neural network. All the networks tested are 3D fully convolutional networks, which represent a subset of CNN architectures. The choice was made to evaluate CNNs as they are by far the most common network architectures in the lung image segmentation literature[^1^](#_ENREF_1). Here, we focused on 3D CNNs due to the volumetric nature of features present in hyperpolarized gas MRI, lending itself towards analysis in a 3D view.

***CNN loss function -*** Loss functions, or cost functions, are used to optimize the network. The loss function dictates how a network’s weights and biases are updated for a given training pass; therefore, they represent a key component to any neural network and are important for generating accurate segmentations.

***Pre-processing -*** Pre-processing is defined as an action taken to modify the training and testing images before they are passed to the network architecture. Pre-processing is often used to enhance features of the image, to accentuate the differences between foreground and background voxels or to remove noise from the image. Numerous studies in the DL-based MRI segmentation literature have employed several pre-processing strategies, including normalization, denoising and N4 bias correction[^1^](#_ENREF_1).

Each experiment trained a CNN for 30,000 iterations on an NVIDIA Tesla V100 graphical processing unit (GPU). Performance was assessed at intervals of 5000 iterations to determine the optimal number of iterations for each network. Shapiro-Wilk tests were performed for each experiment to determine normality and appropriate parametric or non-parametric statistical tests were conducted accordingly.

**Fully convolutional network architectures**

The first parameterization experiment compared the following four 3D CNNs:

- ***VNet:*** A 3D fully convolutional neural network trained end-to-end on volumetric MRI. The network consists of convolutional compressions and subsequent decompression stages until the image is the original size. Each operation is conducted with valid padding. Convolution operations decrease in size from 5x5x5 with a stride of 1 to 2x2x2 with a stride of 2[^2^](#_ENREF_2). The network uses a spatial window size of [96, 96, 32] and a batch size of 6.
- ***Dense VNet:*** Similar to VNet, this CNN employs convolution and deconvolution operations[^2^](#_ENREF_2) with the addition of batch-wise spatial dropout, dense feature stacks and an explicit spatial prior[^3^](#_ENREF_3). Similar to the VNet, we used a spatial window size of [96, 96, 32] and a batch size of 6.
- ***nn-UNet:*** The UNet is a common 2D encoder-decoder network [ref]; here, we used a 3D implementation of the UNet modified to reduce memory constraints, allowing 30 feature channels[^4^](#_ENREF_4). Convolution operations vary in size from 3x3x3 to 1x1x1 depending on the layer of the network. The network also makes use of instance normalization. An isotropic spatial window size of [96, 96, 96] was used with a batch size of 2.
- ***HighResNet:*** A 3D fully convolutional neural network containing 20 layers, the first seven of which used 3x3x3 kernels to capture low-level features. Subsequent layers are dilated by either 2 or 4, with the number of kernels increasing from 16 to 64 to capture high-level features[^5^](#_ENREF_5). Every two layers are grouped with residual connections to form a residual block. An isotropic spatial window size of [96, 96, 96] was used with a batch size of 2.

Supplementary Figure S1 displays the results of the four 3D CNNs, showing mean performance on the parameterization testing set. All networks show improved performance as iterations increase for the DSC and average Hausdorff distance at the boundary (Avg HD) metrics. At 30,000 iterations, we compared performance using DSC and Avg HD across the four network architectures. A Friedman test indicated that there was a statistically significant difference between architectures X^2^(4)=50.75, p<0.0001. Pairwise comparisons were conducted with Bonferroni correction for multiple comparisons. Post-hoc analysis using the DSC and Avg HD metrics showed that both the nn-UNet and the VNet significantly outperformed the other networks tested (p<0.05); however, no statistical difference was observed between the nn-UNet and VNet architectures. Consequently, we could not conclude which network generates the most accurate hyperpolarized gas MRI segmentations for the parameterization dataset. Hence, the loss function experiments were performed for both network architectures.


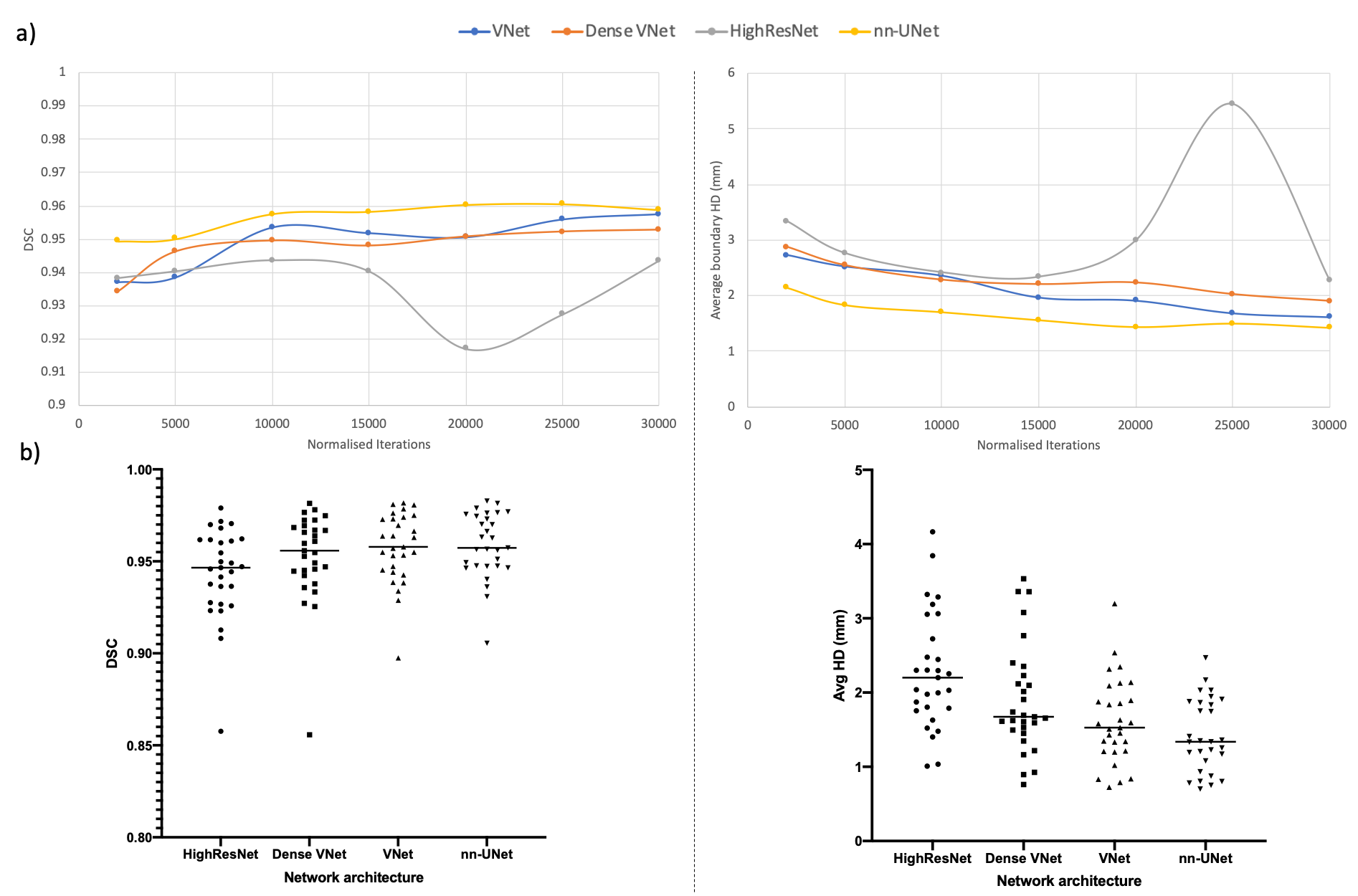


Supplementary Figure S1. Results for the network architecture parameterization experiments showing performance at (a) intervals of 5000 iterations and (b) 30000 iterations for four 3D CNNs in terms of DSC (left) and Avg HD (right).

**CNN loss function**

We compared three common loss functions, namely, the binary cross-entropy (BCE) loss, the dice loss and the Tversky loss. The BCE loss function is defined below:

$$BCE\left( PR,GT \right)= -\frac{1}{N}\sum_{i=1}^{N} {[gt}_{i}\log\left( {pr}_{i} \right)+\left( 1-{gt}_{i} \right)\log\left( 1-{pr}_{i} \right)] ( SEQ "equation" \backslash n \backslash* MERGEFORMAT 1)$$

where $GT=\left\{ {gt}_{i}\in GT \right\}$ denotes the manually-edited ground truth segmentation, $PR=\left\{ {pr}_{i}\in PR \right\}$ the predicted segmentation by the network and $i$ represents the voxel location within the image, which is assumed to have *N* number of voxels.

The Dice loss, which has shown promise in medical image segmentation tasks[^2^](#_ENREF_2) is defined below:

$$Dice\left( PR,GT \right)= \frac{2\sum_{i}^{N} {pr}_{i}{gt}_{i}}{\sum_{i}^{N} {pr}_{i}^{2}+ \sum_{i}^{N} {gt}_{i}^{2}} (2)$$

The Tversky loss[^6^](#_ENREF_6) provides a similar function as the Dice loss; however, it can be weighted to bias the loss function in favor of false positives and false negatives. The Tversky loss is defined below:

$$Tversky\left( PR,GT, \alpha, \beta\right)= \frac{\sum_{i}^{N} {pr}_{i}{gt}_{i}}{\sum_{i}^{N} {pr}_{i}{gt}_{i}+\alpha\sum_{i}^{N} \frac{{pr}_{i}}{{gt}_{i}}+\beta\sum_{i}^{N} \frac{{gt}_{i}}{{pr}_{i}}} (3)$$

where α and β are constants that weight the network's performance towards false positives or false negatives. For this work, we used *α + β = 1,* which reduces the Tversky loss to a set of *F_β_* scores. This has been shown to work well for imbalanced data[^6^](#_ENREF_6). Results for all three loss functions are shown in Supplementary Figure S2.


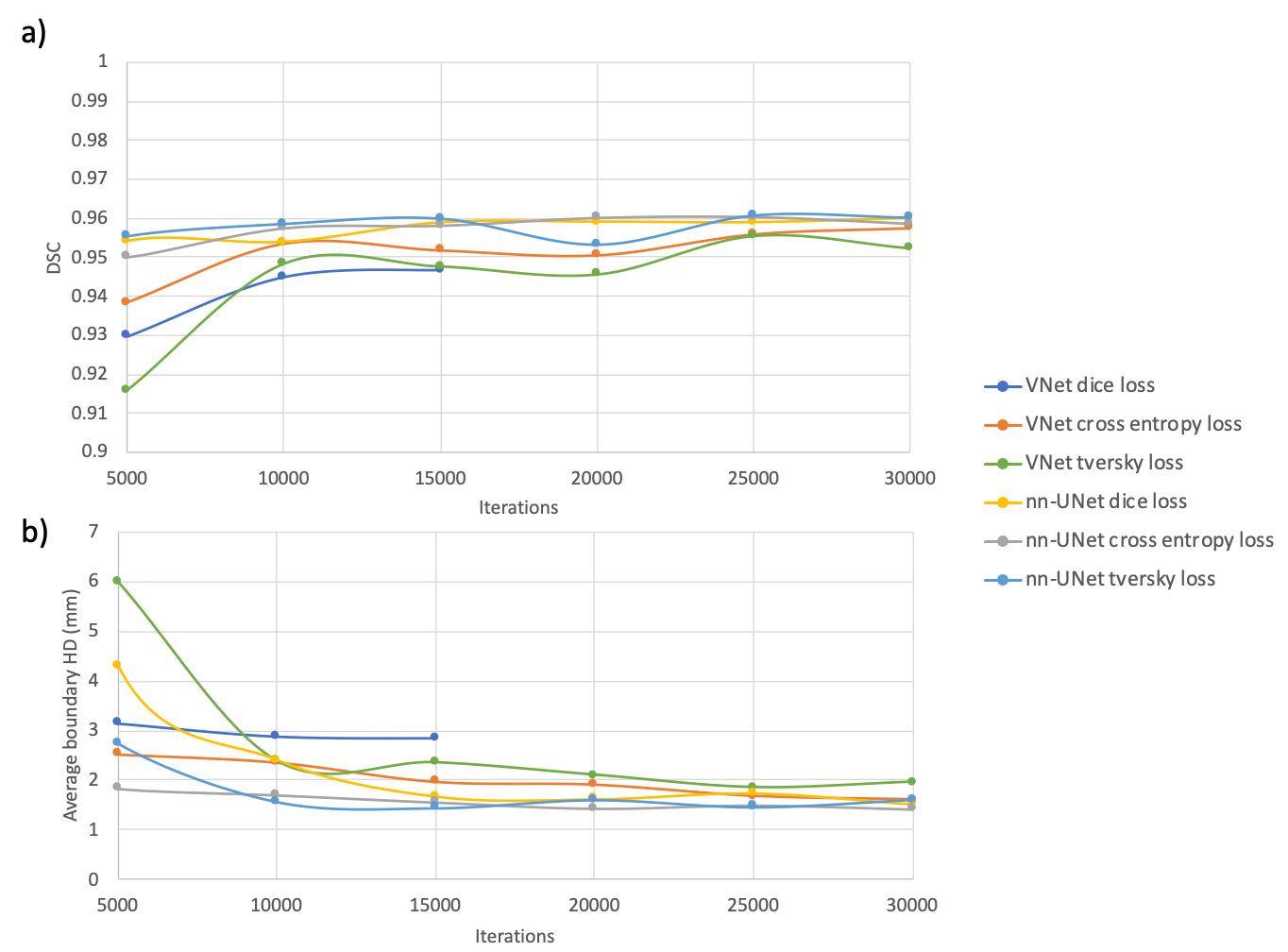


Supplementary Figure S2. Results for both the nn-UNet and VNet architectures using three common loss functions, namely, the binary cross-entropy loss, dice loss and Tversky loss evaluated with a) DSC and b) Avg HD metrics at intervals of 5000 iterations up to 30000 iterations.

Supplementary Figure S2 indicates no meaningful difference at 30000 iterations between any of the loss functions on either network, except for the VNet with Dice loss which exhibited inferior performance. This configuration failed due to an exploding gradient; hence, results are only available up to 15000 iterations. A Friedman test was performed indicating no significant differences between the nn-UNet architectures or the VNet with BCE loss. Consequently, we used the BCE loss and continued to evaluate both the nn-UNet and VNet architectures further for the impact of image pre-processing on performance.

**Pre-processing**

Here, we evaluate the impact of three commonly used pre-processing techniques for hyperpolarized gas MRI, namely, normalization, denoising^[7](#_ENREF_7)^ and N4 bias correction[^8^](#_ENREF_8). In addition, we compared the previous strategies to a combination of all pre-processing techniques and unprocessed images. Supplementary Figure S3a shows that the segmentations produced by the combination of all pre-processing methods perform significantly worse than the other pre-processing methods alone and the images with no pre-processing. Supplementary Figure S3b indicates the performance of each pre-processing method at 30000 iterations.


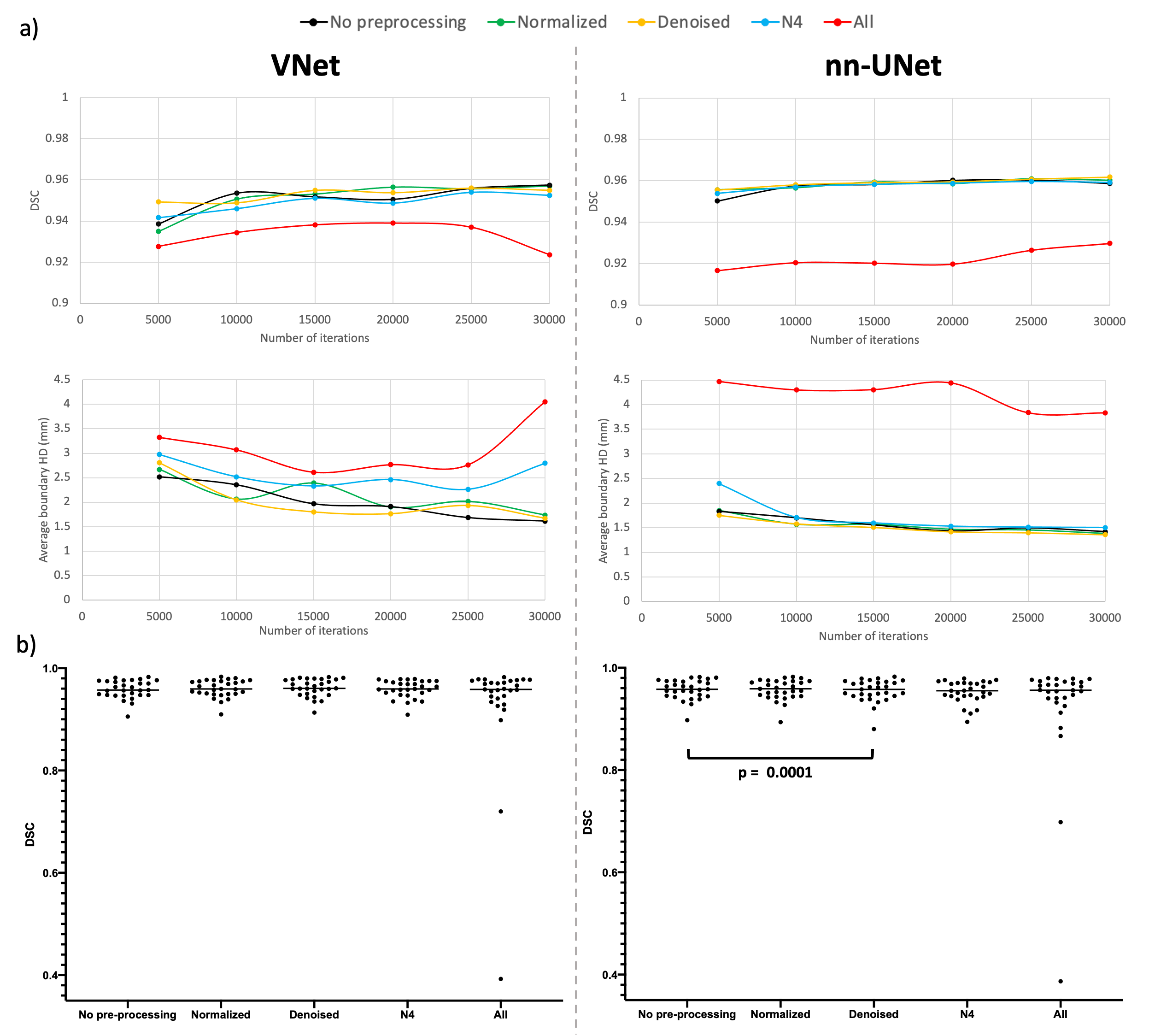


Supplementary Figure S3. a) Results for both the nn-UNet and VNet architectures using common pre-processing strategies evaluated with DSC and Avg HD at intervals of 5000 iterations up to 30000 iterations. b) Results for pre-processing experiments comparing performance at 30000 iterations in terms of DSC.

Friedman tests were conducted for both the VNet (X^2^(5)=23.28, p=0.0001) and nn-UNet (X^2^(5)=25.08, p<0.0001) architectures, indicating significant differences between pre-processing strategies. Pairwise comparisons were conducted with Bonferroni correction for multiple comparisons. No pre-processing was selected as the control group for this experiment. Post-hoc comparisons of the VNet architecture indicated that no pre-processing method provides a statistically significant improvement over scans without pre-processing. Post-hoc comparisons of the nn-UNet architecture indicated that denoising provides a statistically significant improvement over images without pre-processing (p<0.05); no significant differences were observed for the other pre-processing strategies. Accordingly, we compared the nn-UNet denoised model with the VNet unprocessed model using a Wilcoxon signed-rank test and observed that the nn-UNet denoised model exhibited superior performance (p<0.0001).

**Conclusion of parameterization experiments**

Based on the parameterization experiments conducted on a subset of our available data, we determined that for our hyperpolarized gas MRI segmentation problem, the nn-UNet architecture with BCE loss using denoised images generates the best performing segmentations and, therefore, constitutes the optimal configuration for future investigations. Conducting these experiments on a subset of the total data allows for optimization of parameters without introducing potential biases to specific training and testing sets. The following section describes the data split and DL parameters, informed by the above investigations, used in the remainder of this work.

**Supplemental Results**

**2D spoiled gradient-echo ^3^He hyperpolarized gas MRI scans**

**Data acquisition**

We employed a dataset of 2D spoiled gradient-echo ^3^He hyperpolarised gas MRI ventilation scans from 31 patients with either asthma[^9^](#_ENREF_9) (n=12) or cystic fibrosis[^10^](#_ENREF_10) (n=19) acquired at FRC+1L with full lung coverage at 1.5T on a HDx scanner (GE Healthcare, Milwaukee, WI). Helium was polarized on-site to around 25% polarization (GE Healthcare, Amersham, England). Flexible quadrature radiofrequency coils were employed for transmission and reception of MR signals at the Larmor frequency of ^3^He (Clinical MR Solutions, Brookfield, WI) with the following parameters: resolution of ~3x3x10mm^2^, TR/TE equal to 3.6/1.1 milliseconds, field of view of 30-40cm, flip angle of 8º and bandwidth of ±63kHz.

The ^3^He hyperpolarized gas MRI scans differ from the scans used in the primary investigation in terms of both MRI sequence and acquisition parameters.

**Results and Discussion**

Figure S4 shows examples of segmentation quality for one asthma and one CF patient using the combined ^3^He or ^129^Xe DL trained model. The original scans and expert segmentations are included to facilitate comparison. The proposed model accurately excludes subtle and gross ventilation defects in the spoiled gradient-echo hyperpolarized gas MRI scans and excludes airways.


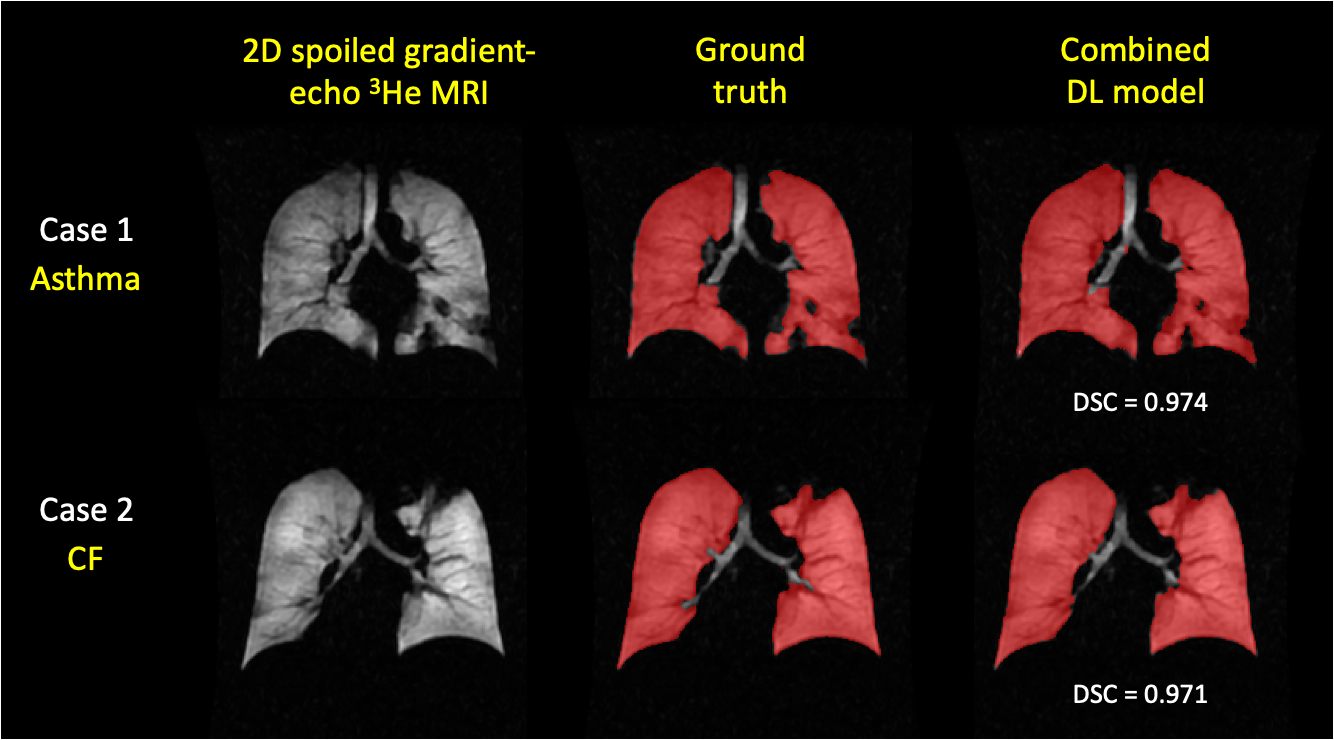


Supplementary Figure S4. Example coronal slices for an asthma and CF case with ground truth segmentations and the segmentations generated by the combined ^3^He and ^129^Xe DL model. DSC values are displayed for reference.

Quantitative results of segmentation performance on the 2D spoiled gradient-echo hyperpolarized gas MRI scans are displayed in Supplementary Table S1. The results indicate that the combined ^3^He and ^129^Xe DL trained model generated segmentations which largely agree with expert ground truth segmentations across a range of metrics.

Supplementary Table S1. Summary of 2D spoiled gradient-echo results.

| **Segmentation method** | **Evaluation metrics: Median (range)** | | | |
| --- | --- | --- | --- | --- |
|  | DSC | Avg HD (mm) | HD95 (mm) | XOR |
| Combined ^3^He and ^129^Xe DL model | 0.965 (0.947, 0.983) | 1.389 (2.030, 0.602) | 4.323 (8.203, 1.934) | 0.069 (0.107, 0.034) |

Abbreviations: dice similarity coefficient (DSC), average boundary Hausdorff distance (Avg HD), 95^th^ percentile boundary Hausdorff distance (HD95), relative error metric (XOR).

These results indicate that the proposed DL model, trained on a combined 3D SSFP ^3^He and ^129^Xe dataset, is generalizable to hyperpolarized gas MRI ventilation scans acquired with a sequence not included within the training set, namely, a 2D spoiled gradient-echo sequence. The segmentation performance on 2D spoiled gradient-echo hyperpolarized gas MRI scans, across all four metrics, are highly similar to the results observed on the 3D SSFP testing set.

**Supplementary Instructions**

**Using the DL trained model**

The model was trained using the Niftynet framework[^11^](#_ENREF_11); instructions are provided below to allow researchers to utilize the trained DL model. The instructions given here will largely follow the instructions given at <https://github.com/NifTK/NiftyNet>.

**Installation**

pip install “tensorflow==1.15.*”

pip install niftynet

We have provided a configuration file that can be used for inference on hyperpolarized gas MRI scans. We highlight the main parameters in the configuration file required for inference that are specific to the primary user.

**Paths**

The user must provide a path to a folder containing the hyperpolarized gas MRI scans and a path to the location to save the outputted segmentations.

path_to_search = #path to hyperpolarized gas MRI scans

save_seg_dir = #path to output location

**Computational resources**

Depending on the computational resources available to the individual researcher, the following parameters will need to be amended:

num_threads = 0

num_gpus = 0

In our investigations, we used a single GPU with two threads.

**Running inference**

The following command can be used to run inference by indicating the path to the configuration file:

net_segment inference -c /path_to_config/config_inference.ini

**Configuration file**

[HPgasMRI]

path_to_search =

filename_contains =

filename_not_contains =

spatial_window_size = (96, 96, 96)

pixdim = (1.0, 1.0, 1.0)

axcodes=(L, P, S)

interp_order = 3

[SYSTEM]

cuda_devices = ""

num_threads = 0

num_gpus = 0

model_dir =

[NETWORK]

name = nonewnet

activation_function = prelu

batch_size = 2

decay = 0.0001

reg_type = L2

volume_padding_size = 10

histogram_ref_file = ./example_volumes/monomodal_parcellation/standardisation_models.txt

norm_type = percentile

cutoff = (0.01, 0.99)

normalisation = True

whitening = True

normalise_foreground_only=True

foreground_type = otsu_plus

multimod_foreground_type = and

queue_length = 10

window_sampling = uniform

[TRAINING]

sample_per_volume = 10

rotation_angle = (-10.0, 10.0)

scaling_percentage = (-10.0, 10.0)

lr = 0.00001

loss_type = CrossEntropy

starting_iter = -1

save_every_n = 1000

max_iter = 150000

validation_every_n = 1000

max_checkpoints = 100

exclude_fraction_for_validation=0.10

exclude_fraction_for_inference=0

[INFERENCE]

border = (0, 0, 1)

inference_iter = 140000

save_seg_dir =

output_interp_order = 0

spatial_window_size = (96, 96, 96)

[SEGMENTATION]

image = HPgasMRI

output_prob = False

num_classes = 2

label_normalisation = False

**References**

1 Astley, J. R., Wild, J. M. & Tahir, B. A. Deep learning in structural and functional lung image analysis. *The British Journal of Radiology* **0**, 20201107, doi:10.1259/bjr.20201107 (2020).

2 Milletari, F., Navab, N. & Ahmadi, S. A. V-Net: Fully Convolutional Neural Networks for Volumetric Medical Image Segmentation. *Proceedings of 2016 Fourth International Conference on 3d Vision (3dv)*, 565-571, doi:10.1109/3dv.2016.79 (2016).

3 Gibson, E. *et al.* Automatic Multi-Organ Segmentation on Abdominal CT With Dense V-Networks. *IEEE Transactions on Medical Imaging* **37**, 1822-1834, doi:10.1109/TMI.2018.2806309 (2018).

4 Isensee, F., Kickingereder, P., Wick, W., Bendszus, M. & Maier-Hein, K. H. in *Brainlesion: Glioma, Multiple Sclerosis, Stroke and Traumatic Brain Injuries.* (eds Alessandro Crimi *et al.*) 234-244 (Springer International Publishing).

5 Li, W. *et al.* On the Compactness, Efficiency, and Representation of 3D Convolutional Networks: Brain Parcellation as a Pretext Task. (2017).

6 Salehi, S. S., Erdogmus, D. & Gholipour, A. *Tversky Loss Function for Image Segmentation Using 3D Fully Convolutional Deep Networks*. (2017).

7 Manjon, J. V., Coupe, P., Marti-Bonmati, L., Collins, D. L. & Robles, M. Adaptive non-local means denoising of MR images with spatially varying noise levels. *J Magn Reson Imaging* **31**, 192-203, doi:10.1002/jmri.22003 (2010).

8 Tustison, N. J. *et al.* N4ITK: improved N3 bias correction. *IEEE Trans Med Imaging* **29**, 1310-1320, doi:10.1109/TMI.2010.2046908 (2010).

9 Tahir, B. A. *et al.* Comparison of CT-based Lobar Ventilation with 3He MR Imaging Ventilation Measurements. *Radiology* **278**, 585-592, doi:10.1148/radiol.2015142278 (2016).

10 Marshall, H. *et al.* Detection of early subclinical lung disease in children with cystic fibrosis by lung ventilation imaging with hyperpolarised gas MRI. *Thorax* **72**, 760, doi:10.1136/thoraxjnl-2016-208948 (2017).

11 Gibson, E. *et al.* NiftyNet: a deep-learning platform for medical imaging. *Comput Methods Programs Biomed* **158**, 113-122, doi:10.1016/j.cmpb.2018.01.025 (2018).
